# Atmospheric rivers fueling the intensification of fog and haze over Indo-Gangetic Plains

**DOI:** 10.1038/s41598-022-09206-9

**Published:** 2022-03-24

**Authors:** Shivali Verma, Muvva V. Ramana, Raj Kumar

**Affiliations:** Land and Atmospheric Physics Division, Earth and Climate Science Area (ECSA), National Remote Sensing Centre (NRSC), Indian Space Research Organization (ISRO), Balanagar, Hyderabad, 500 037 India

**Keywords:** Atmospheric science, Climate change, Environmental impact

## Abstract

Indo-Gangetic Plains (IGP) experiences persistent and widespread rise of fog and haze during the winter season. This has been attributed to the rise in pollution levels and water vapor, but the reason for enhancement in latter is not clear yet. We detect moisture incursion from Arabian Sea, a phenomenon called atmospheric rivers (AR), land-falling intermittently along 12–25° N corridor of the west-coast of India during winter; using satellite and reanalysis data. The total vertically integrated horizontal water vapor transport in AR-landfalls ranging from 0.7 × 10^8^ to 2.2 × 10^8^ kg/s; nearly five-orders of magnitude larger than the average discharge of liquid water from Indus River into Arabian Sea. These AR events are playing prominent role in enhancing water vapor over IGP region by 19 ± 5%; in turn fueling the intensification of fog and haze through aerosol-water vapor interaction. We found that AR events enhanced aerosol optical depths over IGP by about 29 ± 13%. The progression of moist-laden winds in ARs onto Himalayan Mountains contributes to the precipitation that explains the observed rise in the extreme flow of western Himalayan Rivers in winter. We conclude that these ARs likely contribute to the decline of snow albedo as pollution-mixed-ARs encounter Hindukush-Karakoram-Himalayan mountain region.

## Introduction

A dense haze and fog blankets nearly the whole Indo-Gangetic plains (IGP) during the winter months of December-January each year^1,2^. In general, fog formation over IGP and North-Indian region is a common feature in the winter season every year with around 60% of days being foggy^3^. However, sudden increase in spatiotemporal extent of fog and haze has been reported in recent years^4–6^. A significant decline in visibility^7^ and gradual increase in number of fog and dense fog days^3^ over IGP was reported using ground-based surface visibility measurements over three-decades. Satellite and reanalysis datasets also show an increasing trend in the number of hazy days at a rate of ~ 1.7 days/year over IGP using 2003–2017 data^8^.

IGP is among few regions of the world with a thick layer of anthropogenic aerosols during the winter season^9^. In order to understand the wintertime aerosol properties across the IGP, the Indian Space Research Organization under the Geosphere Biosphere Programme conducted field campaigns^10–12^ in the IGP during December 2004. These measurements reveal the enhancement of extinction coefficient by almost 2–3 times below 0.2 km altitude on hazy and foggy days compared to non-foggy days^10^. The concentrations of elemental carbon, organic carbon, and water-soluble organic carbon have reported nearly 30% increase during fog and haze events; whereas inorganic constituents are 2–3 times higher than those during clear days over IGP during the winter season^12^. To understand the fog formation and dissipation over IGP for better nowcasting and forecasting, Ministry of Earth Sciences, India conducted an intensive field campaign, Winter Fog Experiment^3^, during winter season of 2015–16 at New Delhi. These measurements helped to simulate the life cycle of dense fog events^3^, which further improved the prediction of fog^13,14^.

Previous studies have focused on the detection of fog^15–18^, measuring the optical properties of aerosol and fog^19,20^, fog forecast^21,22^, implications of the growing extent fog^22,23^ and the persistence of fog^24^. However, there is still a lack of scientific explanation for the reason behind the spatiotemporal intensification of fog and haze across the IGP region. Increasing atmospheric pollution combined with sufficient moisture (thus the availability of cloud condensation nuclei) favor the fog and haze formation^3,25^. Enhanced aerosol loading over IGP is well known, but the water vapor needed for intensification of fog and haze in IGP during winter is not known. This is further leading to a challenge in simulating the aerosol size, properties and fog prediction over this region^26,27^. In general, cool and dry conditions prevail over IGP during the winter season. The eastward-moving synoptic weather systems through 25–40° N, 60–80° E region^28^ during winter, known as western disturbances^29^, bring occasional precipitation over this region. These synoptic weather systems provide moisture and contribute to low temperatures, which in turn favor the development of cold waves. However, IGP experienced a decline (or no significant trend) in the frequency of Western Disturbances^30^. In this study, for the first time, we present intense moisture incursion from Arabian Sea onto the west coast of India through 12–25° N corridor during December to January each year using remote sensing (2013–2020) and reanalysis (2000–2020) data. We link the finding to fog and haze intensification over IGP region as well as the recent hydro-climate signal observed over western Himalayas in recent decades.

### Atmospheric rivers

**Figure 1:**
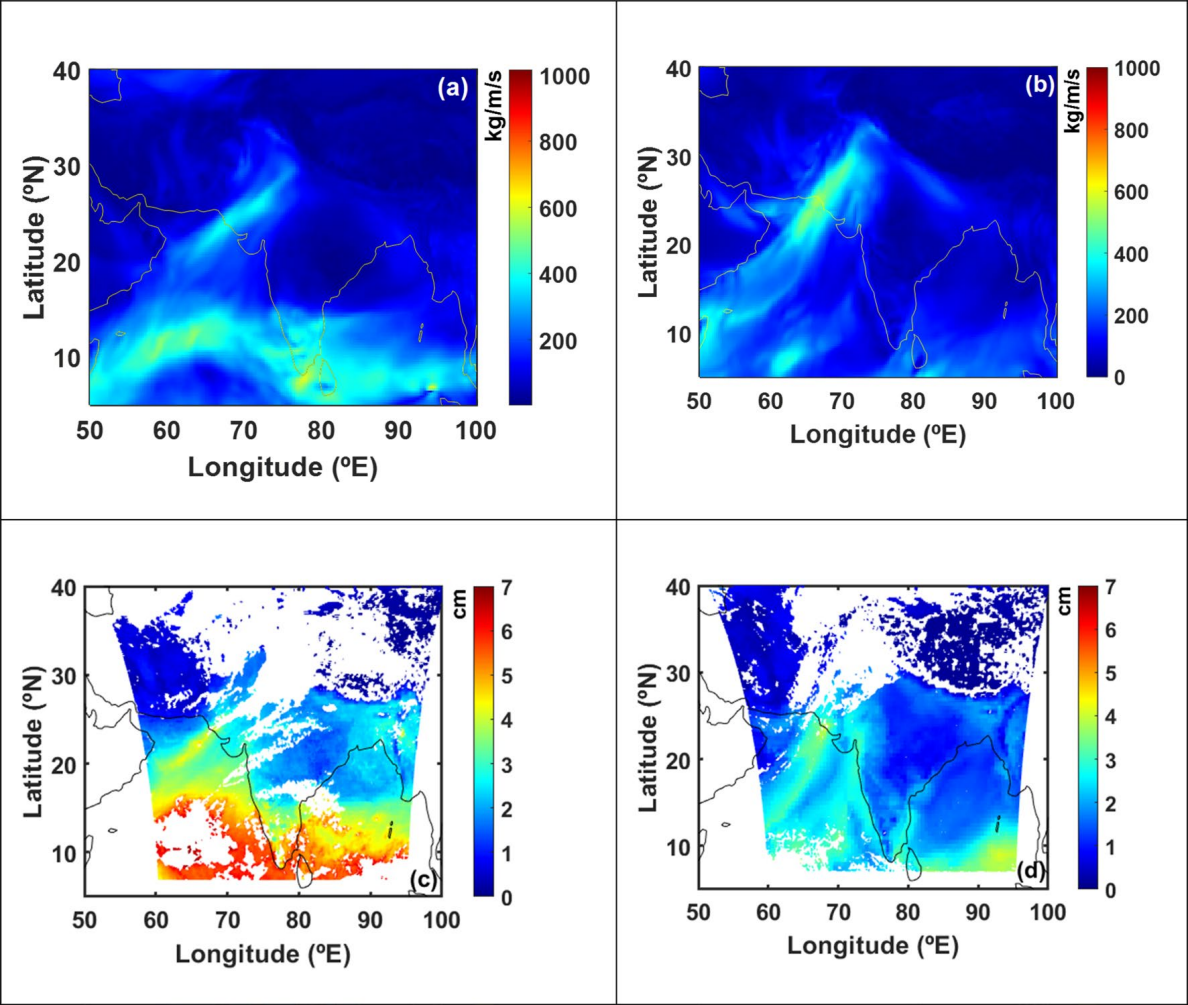
Atmospheric rivers (AR) over the west coast of India. Integrated horizontal water vapor transport (IVT, in Kg/m/s) values from reanalysis data over India and surrounding Ocean on (**a**) Dec 9, 2015 at 09UTC and (**b**) Jan 12, 2020 at 17UTC showing ARs at west coast of India. Satellite measured precipitable water vapor (PWV, in cm) during the AR events on (**c**) Dec 9, 2015 at 09UTC and (**d**) Jan 12, 2020 at 17UTC. White color in figures (**c**) and (**d**) indicate non-availability of data. Figure is generated using MATLAB R2020a software available at http://in.mathworks.com/products/matlab/. (License no: 1103382).

The vertically integrated horizontal water vapor transport (IVT) values on Dec 9, 2015 (at 09UTC) and Jan 12, 2020 (at 17UTC) over India and surrounding oceanic regions are shown in Fig. 1a,b, using reanalysis data. These figures illustrate southwesterly moisture transport from Arabian Sea onto the west coast of North India centred at around 24.66° N 67.20° E and 24.21° N 67.13° E. A narrow and elongated structure of intense IVT features of around 2000 km in length and 500 km in width having IVT magnitude as high as 400 kg/m/s in the core. The description of atmosphere river (AR) characteristics^31^ defined by the shape, extent and axis of horizontal moisture transport, that make landfall over the long narrow corridors, imply the presence of ARs over the coastal region of North India during the winter season. This data is also complemented by satellite measured precipitable water vapor (PWV), shown in Fig. 1c,d, where PWV values are around 4 cm in the ARs core. Both IVT and PWV depict intense moisture transport in this region; however, IVT values show a better depiction of AR than PWV values. This is because of the fact that PWV represents the features associated with water vapor concentrations in the atmosphere, while the IVT captures the transport of water vapor (i.e., water vapor combined with winds) in the atmosphere. The total IVT (TIVT) within the ARs for Dec 9, 2015 and Jan 12, 2020 (shown in Fig. 1a,b), are 1.48 × 10^8^ kg/s and 1.77 × 10^8^ kg/s respectively (see Methods section for TIVT estimation and Supplementary Fig. S1). The duration of these ARs, from its genesis in the Arabian Sea to its landfall; are about 76 and 63 h respectively.

### AR-landfall frequency and characteristics

**Figure 2:**
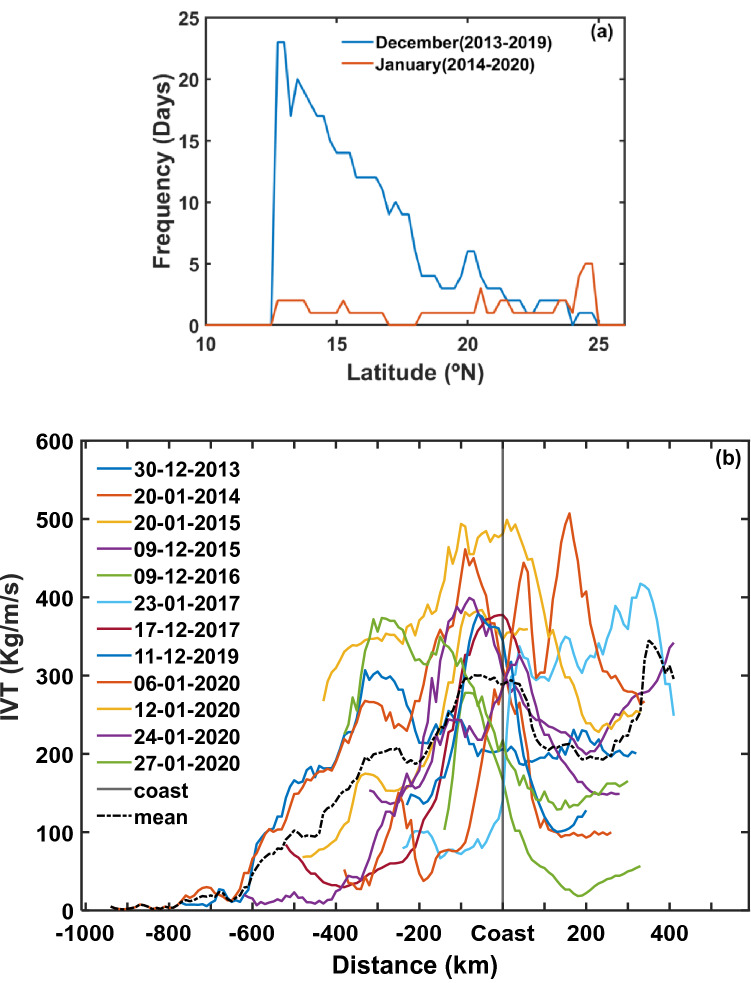
AR-landfall frequency along the west coast of India. (**a**) Frequency of AR-landfalls along the west coast of India during 2013–2020 estimated based on IVT and PWV values. (**b**) The AR-landfall transects along the west coast of India correspond to the maximum IVT as close as possible to the midpoint of each transect on the coast during 2013–2020. The negative and positive values on the horizontal distance scale represents respectively offshore and inland sides of AR axis with reference to the coast. Black dashed line represents the mean IVT of all ARs.

AR-landfall causes an enhancement of IVT along the coast depending upon where AR axis intersects the coastline. The frequency of daily mean IVT greater than 250 kg/m/s and PWV greater than 4 cm along the west coast of India from 5 to 30° N for December and January of 2013–2020 are shown in Fig. 2a. The AR landfalls over this region are contributing to the more frequent occurrence of moisture transport predominantly occurring between 12 and 25° N region. It is observed that frequency and all possible entry locations of ARs into India decreases abruptly south of 12° N and to the north of 25° N. The core of AR activity is maximum around 12° to 15° N in December, whereas it uniformly distributed in January.

Further, we detected AR events that made landfall at west coast of India during December and January months of the study period (2013–2020). The AR patterns that persisted for at least 48 consecutive hours and having at least 1000 km long contiguous structure in their entirety (offshore and inland combined) are presented here. The location corresponding to the IVT and PWV maximum along the west coast of India is considered as the central land-falling location of AR. The offshore-inland axis of each AR at coastal coordinates, shown in Fig. 2b, was obtained based on maximum IVT points within AR and then a line drawn following these points. These transects are mostly found over the west coast of North India between 12 and 25° N in a location that is observed to experience high IVT conditions during the winter months. The observed magnitude of transect values of IVT at the coast are greater than 200 kg/m/s. These moisture structures are typically having width-to-length ratios of 0.1 to 0.5 with TIVT values ranging from 0.68 × 10^8^ to 2.16 × 10^8^ kg/s (Supplementary Table S1). The mean TIVT of all AR events shown in Fig. 2b is 1.62 × 10^8^ ± 0.23 × 10^8^ kg/s. The average discharge of the Indus River^32,33^ water into the Arabian Sea is around 3.0 × 10^3^ m^3^/s; for comparison, the average water vapor flux in the observed AR events is nearly five-orders of magnitude more than the liquid water discharge into the Arabian Sea from the Indus River.

The daily mean IVT values added over 12° to 25°N for December and January months show a steady rise of IVT at 1.7%/year and 0.1%/year respectively during 2000–2020 time period (Supplementary Fig. S2). Since IVT has been widely used to track ARs^31^, the steady increase in IVT reflects the increase in AR landfall frequency (or moisture transport) onto the coast. The possible explanation for the observed trends in land-falling ARs across the west coast of India is due to increase in lower atmospheric moisture content and changes in atmospheric circulation patterns over this region. Our analysis shows an increasing trend in integrated water vapor (IWV) over most of the Arabian Sea during the past 20 years from 2000 to 2020 for December and January months (Supplementary Fig. S3). The rising trends in global specific humidity have been observed in the past decades^34–37^ and are projected to continue to increase over the twenty-first century^37,38^. Concurrently, meridional wind component show an increasing trend over this region for the same time-period than zonal wind component (Supplementary Fig. S4). Recent studies have reported the pole-ward migration of atmospheric flow in the Northern Hemisphere during fall and winter^39,40^. Thus, it is likely that increase in moisture availability in combination with a meridional intensification of the flow pattern during recent decades support the increase in ARs frequency along the west coast of India.

### Impact of ARs

**Figure 3:**
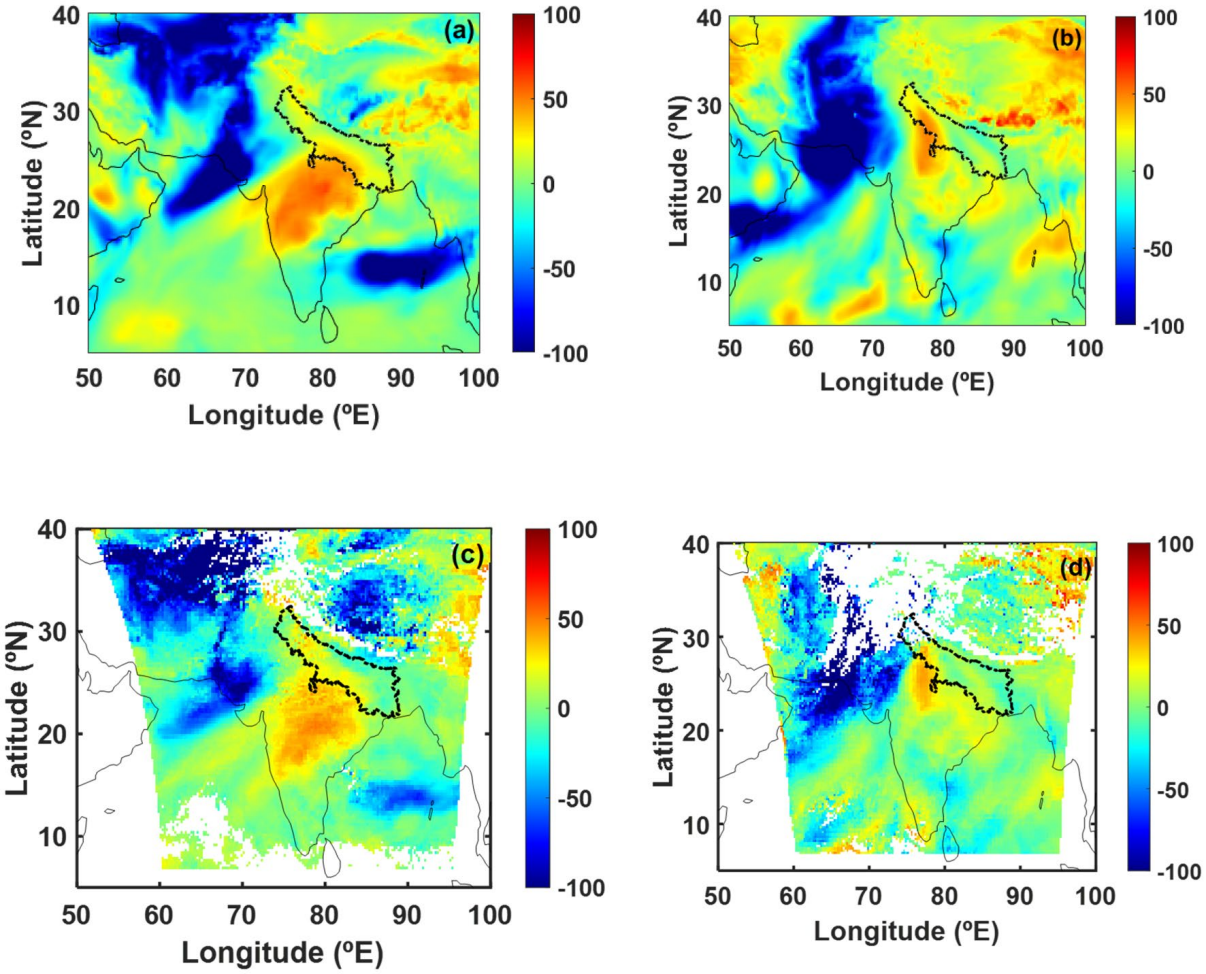
Fractional contribution of AR to the water vapor amount over IGP region. Contribution of AR to the integrated water vapor (IWV) using reanalysis data over IGP on (**a**) Dec 10, 2015 and (**b**) Jan 13, 2020. Contribution of AR to precipitable water vapor (PWV) using satellite data over IGP on (**c**) Dec 10, 2015 and (**d**) Jan 13, 2020. Black dashed line in the figure represents the IGP region. White color in figures (**c**) and (**d**) indicate non-availability of data. Figure is generated using MATLAB R2020a software available at http://in.mathworks.com/products/matlab/. (License no: 1103382).

While AR-landfall has not been investigated previously over IGP region during winter season, it is hereby shown to be a critical factor in intensification of fog and haze. The fractional contribution of AR to the water vapor amount over IGP is estimated (Methods section), as AR stretches from the coastal region (on the days shown in Fig. 1) into the IGP region (on consecutive days). Figure 3a,b shows the fractional contribution of moisture-laden winds in AR to the column integrated water vapor amount over the IGP region on Dec 10, 2015 and Jan 13, 2020 respectively using reanalysis data (Supplementary Fig. S5). We observe that the moisture in AR enhanced the water vapor content over the IGP region by a regional-mean value of 19 ± 10% and 18 ± 13% respectively (Supplementary Fig. S6). Satellite-measured precipitable water vapor also supports the large enhancement in water vapor over IGP indicating similar increase as shown in Fig. 3c,d for the same days. The close agreement between reanalysis and satellite data validates the inferred AR contribution to the enhancement in water vapor amount over IGP region. The ARs impact is felt over IGP region owing to its topography as it is a transitional zone between Himalayas of the north and peninsular India of the south with an average elevation of about 200 m above mean sea level. Satellite images on these respective days also confirm increase in fog and haze over IGP region (Supplementary Fig. S7).

**Figure 4:**
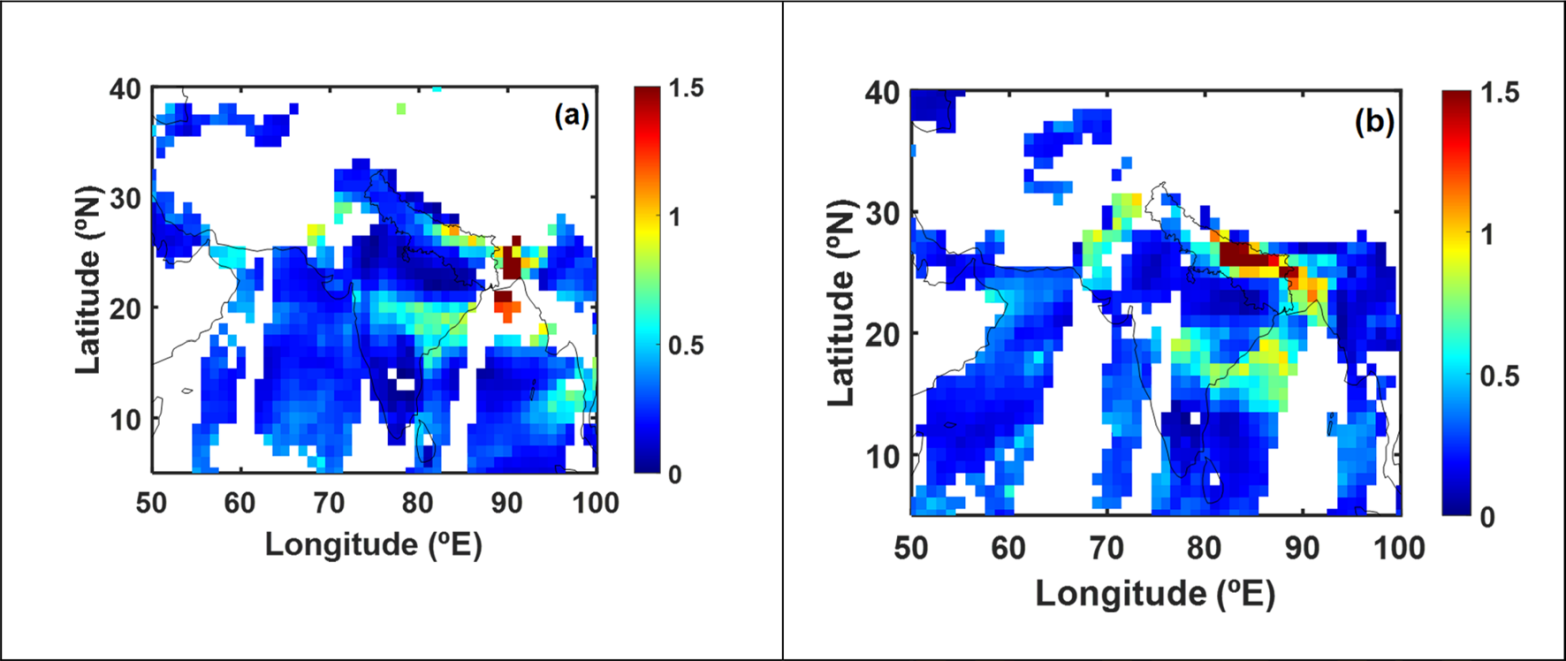
AR-driven changes in aerosol optical depths (AOD) over the Indo-Gangetic Plains for an event on Jan 24–25, 2020. The distribution of AOD on two consecutive days, (**a**) AOD on Jan 24, 2020 at 10.30 local time and (**b**) AOD on Jan 25, 2020 at 10.30 local time, which corresponds to before and after passage of AR event over IGP. The mean AOD values over IGP for Jan 24, 2020 and Jan 25, 2020 are 0.36 ± 0.25 and 0.92 ± 0.66 respectively. White color in figures represents non-availability of data. Figure is generated using MATLAB R2020a software available at http://in.mathworks.com/products/matlab/. (License no: 1103382).

In order to quantify the impact of AR on aerosols, satellite retrieved aerosol optical depths (AOD) over IGP for the AR event on Jan 24–25, 2020 is depicted in Fig. 4. The AR event that made landfall on west coast of India on Jan 24, 2020 has moved inland with TIVT of 1.52 × 10^8^ kg/s (mean IVT value of 247 ± 186 kg/m/s) and reached IGP on Jan 25, 2020. The water vapour flux from AR enhanced the water vapour content over IGP by 36% and AOD almost by a factor of 2 (Supplementary Table S2). Concurrently, fog enhancement is seen on Jan 25, 2020 relative to Jan 24, 2020 (Supplementary Fig. S8). Thus, the AR-driven enhancement in water vapour over this highly polluted location caused aerosols to undergo aerosol-water vapor interactions^41,42^, leading to the intensification of fog and haze. In general, the detected AR events are observed to enhance the water vapour amount and AOD over IGP by 19 ± 5% and 29 ± 12% respectively (Supplementary Table S3 and S4). Further, a recent study^43^ also reported that aerosols in the IGP were observed to have enhanced water uptake ability. Furthermore, hygroscopic experiments carried out over highly polluted locations of the IGP have also reported the hygroscopicity of aerosols and their ability to serve as cloud condensation nuclei (CCN) under both freshly emitted primary and secondary aerosols^44–46^. A recent observational study^47^ has also reported an enhancement of aerosol size by around 1.34 times at 85% relative humidity and the aerosol hygroscopic growth reported to contribute up to 40% to the observed increase in the aerosol optical depth over IGP during the winter season^41^. Thus, the AR-induced enhancement in water vapor interacts with the prevailing atmospheric aerosols over the IGP region and triggers the aerosol hygroscopicity leading to the intensification of fog and haze.

**Figure 5:**
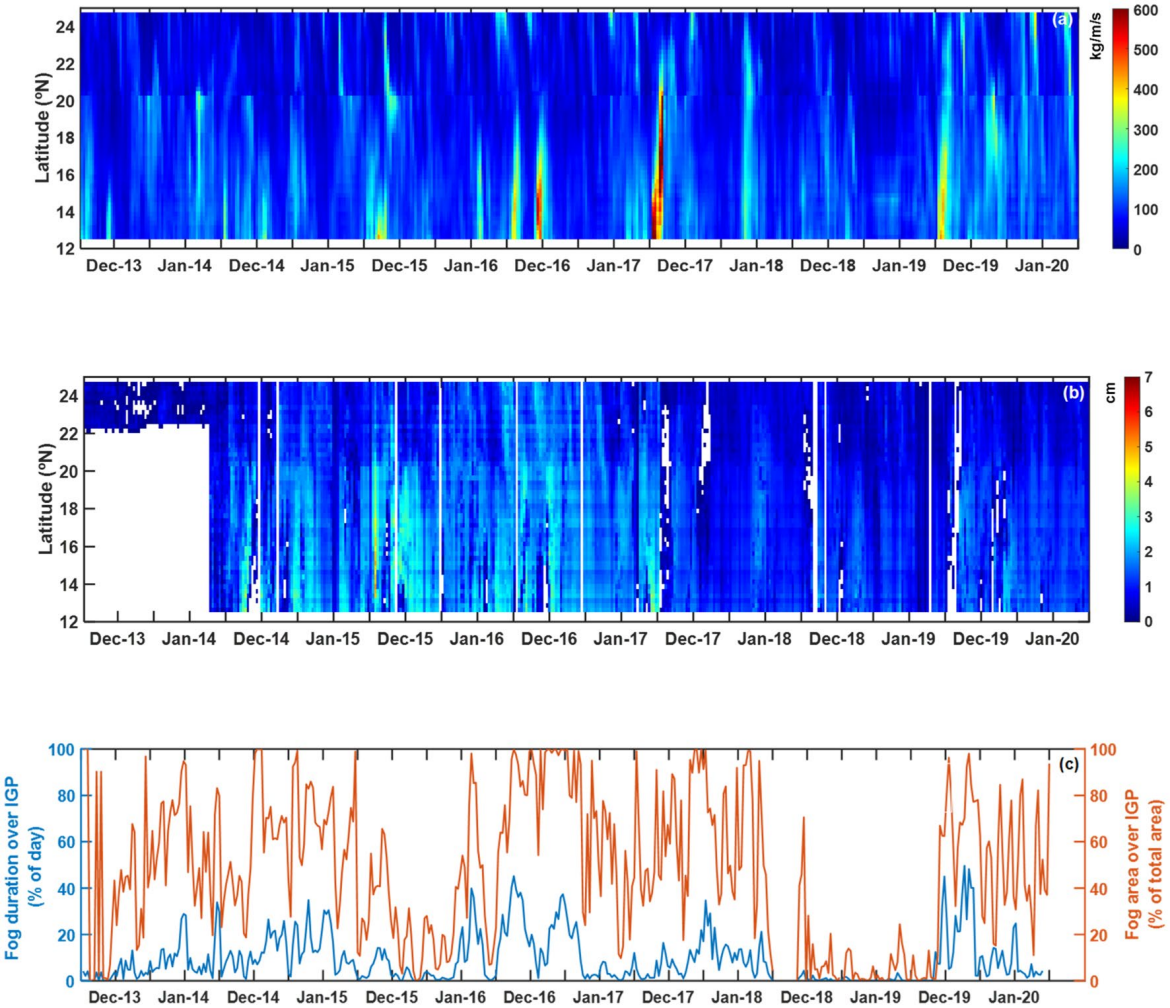
Concomitant of AR-landfall to the intensification of fog over IGP. Temporal variation of (**a**) IVT (in Kg/m/s) and (**b**) PWV (cm) along the west coast of India for December and January months during 2013–2020. (**c**) Temporal variation of fog duration (in blue colour) and spatial extent of fog (in orange color) over IGP region. White color in figure (**b**) indicates non-availability of data.

Further, the close link between AR induced moisture enhancement and fog intensification over IGP region is depicted in Fig. 5. The time series of daily variation of IVT and PWV values along the west coast of India during 2013–2020, shown in Fig. 5a,b respectively, clearly depicts the presence of AR-landfalls. The corresponding time series of satellite retrieved spatial and temporal variation of fog, shown in Fig. 5c over IGP region, illustrate an enhancement of fog in concurrence with AR events. Thus, this suggests that the proper representation of AR phenomenon and associated processes in models may help to simulate the relative humidity accurately over this region, which is reported to be the main challenge in simulating aerosol properties and hygroscopic growth^26,27^. Fog over IGP observed to increase by 1 day/year temporally and approximately by 1 deg spatially during 2013–2019 time periods (Supplementary Fig. S9).

The meteorology and hydrological changes over Himalayas have not been analysed in the context of ARs and it is our hypothesis that the observed ARs in this region during the winter season might be contributing a large fraction of precipitation when moist-laden winds in AR encounters the Himalayan mountain terrain. Recent observational study^48^ reported the rise in the occurrence of extreme flow of western Himalayan Rivers during the winter season, which is attributed to the increased precipitation has doubled. We postulate this ambiguous origin of precipitation during winter to ARs as cross-barrier moisture contributes to the precipitation^49^. Overall, the meteorology and hydrological impacts of AR over Himalayas needs to be explored, especially in the context of a changing global climate system. Such study may provide insights into the processes and mechanisms behind extreme weather conditions, such as flash floods and glacial lake outbursts, over this region and thereby support greater emergency preparedness.

## Data and methods

### Satellite data

Indian Geostationary Satellite, INSAT-3D, retrieved precipitable water vapor (PWV)^50^ and fog mask^17^ data are used in this study. INSAT-3D provides data at 30min interval, covering − 10° to 45° N; 44.5° E to 105.5° E region^17,50^. The PWV data is available at hourly interval and at a spatial resolution of 0.04° × 0.04° from 2013 to 2020 over the Indian region under cloud-free conditions. The uncertainties^50^ in PWV values are less than 2mm. The fog mask^17^ product is available under cloud-free conditions at every 30min temporal interval and at a spatial resolution of 0.04° × 0.04°. The reported accuracy^18^ of fog detection in terms of percentage of detection over IGP for December and January months is around 85%. The total duration of fog presence per day (i.e., hours per day) at pixel level (i.e., 0.04° ×  0.04°) is estimated on daily basis from the respective 30 min fog mask data. Spatial and temporal analysis of fog over IGP for winter months of December and January, for a period of 7 years from 2013 to 2020 was carried out using this fog mask product. Fog duration in each day was computed by finding total number of fog hours in a day in each pixel and then added up for IGP region. This was then normalised by total number of pixels in IGP times 24 h. Similarly, area was calculated by finding fog pixels where fog persisted for more than 5 h in a day. Each pixel has a spatial resolution of nearly 4 km × 4 km, so the total area was normalised by the total area of IGP, which is approximately 6,65,952 sq km. Also, MODIS-Terra (10.30LST) and MODIS-Aqua (13.30LST) retrieved aerosol optical depth (AOD at 550nm) data are used in this study. This data is obtained from collection 6.1, quality assured L3 data of daily global aerosol optical depth (at 550nm) from MODIS (MOD08_D3 and MYD08_D3) on-board Terra and Aqua^51,52^, during 2013–2020. MODIS retrieves AOD with an estimated uncertainty^53^ of ± 0.05 ± 0.20 × AOD over the land. The data set used is at spatial resolution of 1°×1°. Mean values of AOD and corresponding standard deviations over IGP is computed.

### Reanalysis data

The hourly atmospheric reanalysis data from the European Centre for Medium-Range Weather Forecasts (ECMWF), called ERA5 (5th generation ECMWF Re-Analysis)^54^ are used in this study for computing vertically integrated horizontal water vapor transport (IVT) and Integrated water vapor (IWV) over India and surrounding oceanic regions. ERA5 provides atmospheric, land and oceanic variables data on a 0.25° × 0.25° spatial resolution at 37 pressure levels, from the surface up to 80 km altitude^54^. The IVT and IWV in an atmosphere column are computed as:

$$IVT = \sqrt {\left(\frac{1}{g}\mathop \smallint \limits_{Ps}^{Pt} qu\, dp\right)^{2} + \left( {\frac{1}{g}\mathop \smallint \limits_{Ps}^{Pt} qv\, dp } \right)^{2} } ;\;IWV = \frac{1}{g}\mathop \smallint \limits_{Ps}^{Pt} q\, dp.$$

where g is the acceleration due to gravity (m/sec^2^), q is the specific humidity (kg/kg), u and v denote respectively zonal and meridional components of wind (m/sec), Ps surface pressure (bottom most layer of the model, 1000 mb), Pt an upper- atmospheric reference pressure (500 mb), and dp is the pressure difference (mb) between the two adjacent levels. IVT is given in kg/m/s and IWV in Kg/m^2^. The Total IVT (TIVT) within the atmospheric river (AR) is calculated as the horizontal integral of IVT across the AR transect perpendicular to the direction of mean vapor transport^55,56^,

$${\text{TIVT}} = \int {{\text{IVT}}_{{{\text{transect}}}} {\text{dw}}}$$

where IVT_transect_ is the component of IVT normal to the AR transect, and the integration is over all pixels that form the AR transect. The lateral edges of ARs is identified by checking if the IVT value surpassed a given threshold defined locally as the 85th percentile value^57^. To understand the spatiotemporal change in IVT, IWV, zonal and meridional winds, linear trend analysis was conducted over 20-year time period.

### AR contribution to water vapor over IGP

The fractional contribution (f_m_) from moisture-laden transport in AR to the water vapor amount over IGP is computed^58^ as the ratio of the uptake amount of water vapor (Δq_m_) to the water vapor in the air (q_n_), i.e., $f_{m} = \frac{{\Delta q_{m} }}{{q_{n} }}$. Here, Δq_m_ represents the diurnal-change in IWV and PWV values over IGP; which is computed by taking the difference between the current day value (i.e., after AR advances to IGP region) and the previous day value, and q_n_ is the diurnal mean IWV or PWV value on the current day. In this study, fractional contribution (f_m_) is computed at pixel level and expressed in percentage (i.e., f_m_ × 100 to express in percent contribution).

## Supplementary Information

Supplementary Information.

## Data Availability

The satellite data (2013–2020) and reanalysis data (2000–2020) used in the present analysis are freely available online from MOSDAC (https://www.mosdac.gov.in/), MODIS-Terra/Aqua data (2013–2020) from (https://earthdata.nasa.gov/) and ERA5 (https://cds.climate.copernicus.eu/cdsapp#!/home) websites respectively.
