# Supplementary material for: Atmospheric rivers fueling the intensification of fog and haze over Indo-Gangetic Plains
Source: Sci Rep. 2022 Mar 24;12:5139. doi: 10.1038/s41598-022-09206-9 (PMC8948212; doi:10.1038/s41598-022-09206-9)
Supplement: Supplementary file 1 — Supplementary Information. [file 41598_2022_9206_MOESM1_ESM.docx]

**Supplementary Information**

Atmospheric Rivers Fueling the Intensification of Fog and Haze over Indo-Gangetic Plains

**This Supplementary Information includes:**

**Supplementary Figures S1 to S9**

**Supplementary Tables S1 to S4**

| (a) Dec 9, 2015 (at 0900 UTC)   | (b) Jan 12, 2020 (1700 UTC)   |
| --- | --- |
| Length:2,273 km  Width:761 km  Width-to-length Ratio: 0.33  Average IVT: 223 kg/m/s  Total IVT: 1.48 x10^8^ kg/s | Length: 1,997km  Width: 420 km  Width-to-length Ratio: 0.21  Average IVT: 340 kg/m/s  Total IVT: 1.77 x 10^8^ kg/s |

**Supplementary Figure S1**: AR boundary based on 85^th^ percentile value of IVT on (a) Dec 9, 2015 at 09UTC and (b) Jan 12, 2020 at 17UTC. The AR parameters are provided beneath its respective figure. Figures are generated using MATLAB R2020a software available at <http://in.mathworks.com/products/matlab/>. (License no: 1103382).

| 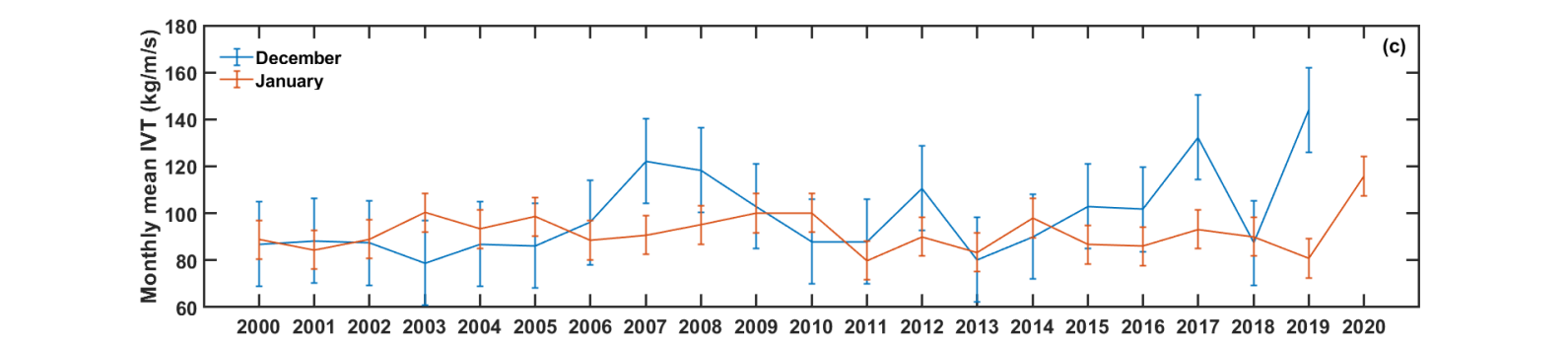 |
| --- |

**Supplementary Figure S2**: Mean IVT and its annual variation added over 12-25°N coastal region for December (in blue color) and January (in orange color) months from 2000 - 2020. The estimated linear trend in IVT for December month is 1.5± 0.2 Kg/m/s/year and for January month is 0.1 ± 0.6 Kg/m/s/year.

Total change in 20 years for December = (1.5 kg/m/s/year * 20 years) / (87 kg/m/s)

= 34%

= 1.7% per year

Total change in 21 years for January = (0.1 kg/m/s/year * 21 years) / (89 kg/m/s)

= 2%

= 0.1% per year

| (a)   | (b)   |
| --- | --- |

**Supplementary Figure S3**: Spatial variation of trends in integrated water vapor (IWV) from reanalysis data for (a) December during 2000 – 2019 and (b) January during 2000 – 2020. Positive values show the regions, where IWV is increasing and negative values indicate those, where IWV is decreasing. Figures are generated using MATLAB R2020a software available at <http://in.mathworks.com/products/matlab/>. (License no: 1103382).

| (a) Trend in **zonal-**component of wind for **December during 2000-2019**  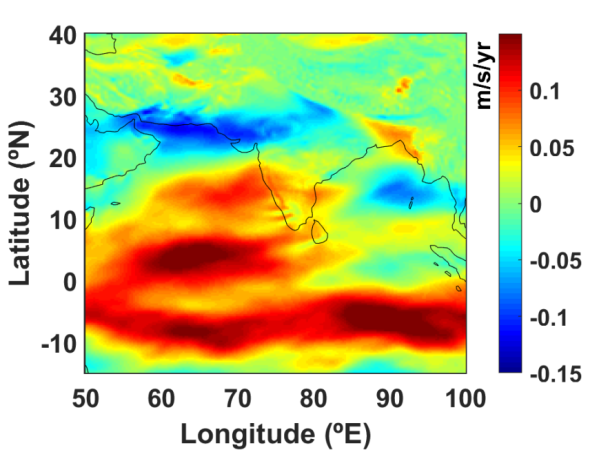 | (b) ) Trend in **meridional-**component of wind for **December during 2000-2019**  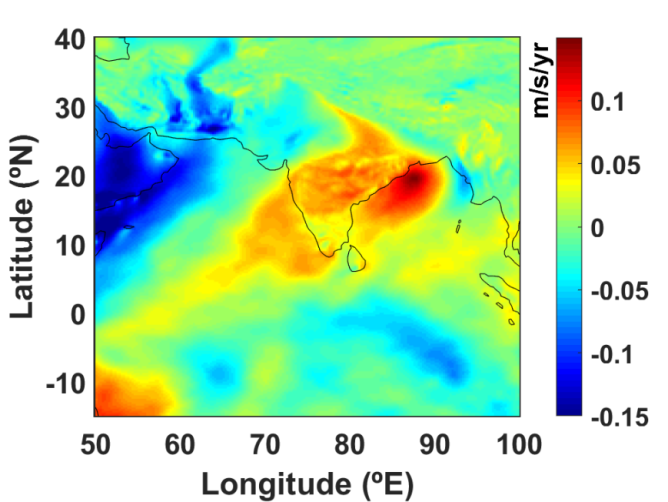 |
| --- | --- |
| (c) Trend in **zonal**-component of wind for **January during 2000-2020**  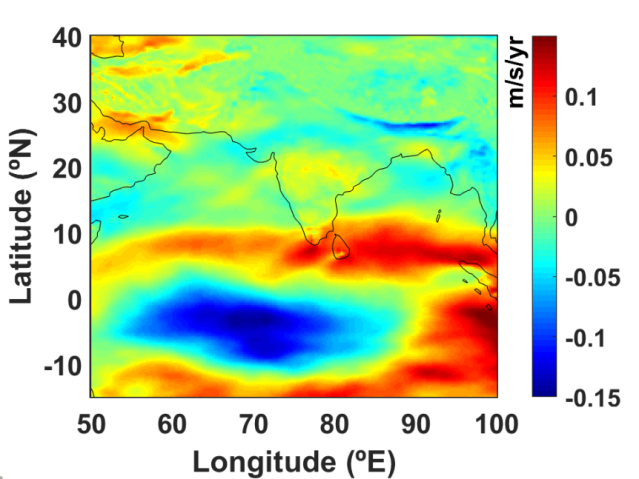 | (d) Trend in **meridional-**component of wind for **January during 2000-2020**  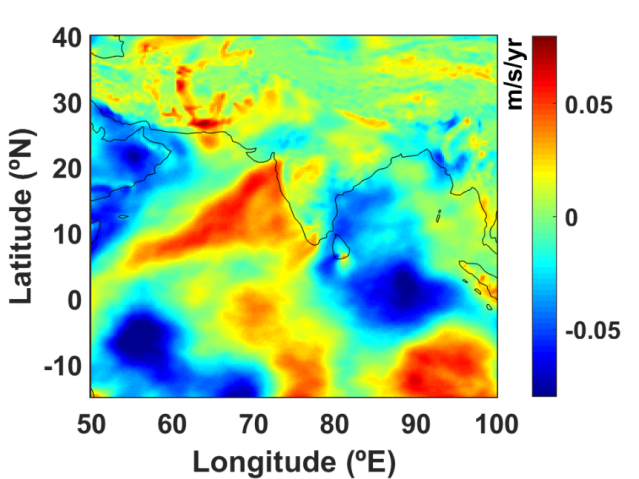 |

**Supplementary Figure S4**: Spatial variation of trends in (a) zonal and (b) meridional components of wind for December during 2000 – 2019. Spatial variation of trends in (c) zonal and (d) meridional components of wind for January during 2000 – 2020. Positive values show the regions, where IWV is increasing and negative values indicate those, where IWV is decreasing. Figures are generated using MATLAB R2020a software available at <http://in.mathworks.com/products/matlab/>. (License no: 1103382).

| **(a)**  **** | **(b)**  **** | **(c)**  **** |
| --- | --- | --- |
| **(d)**  **** | **(e)**  **** | **(f)**  **** |

**Supplementary Figure S5:** Integrated Water vapor (IWV, Kg/m^2^) values over India and surrounding ocean using ERA5 data. (a) Dec 9, 2015 and (b) Dec 10, 2015, (c) percentage change in IWV from Dec 9 to Dec 10, (d) Jan 12, 2020 and (e) Jan 13, 2020 and (f) percentage change in IWV from Jan 12 to Jan 13. Figures are generated using MATLAB R2020a software available at <http://in.mathworks.com/products/matlab/>. (License no: 1103382).

| (a)   | (b)   |
| --- | --- |

**Supplementary Figures S6:** Histogram showing the fractional contribution of AR to the water vapor over IGP in steps of 10% on (a) Dec 10, 2015 and (b) Jan 13, 2020. This frequency analysis pertains to the IGP region, as shown in Figure 3(a) and 3(b) with dotted line.

| (a)  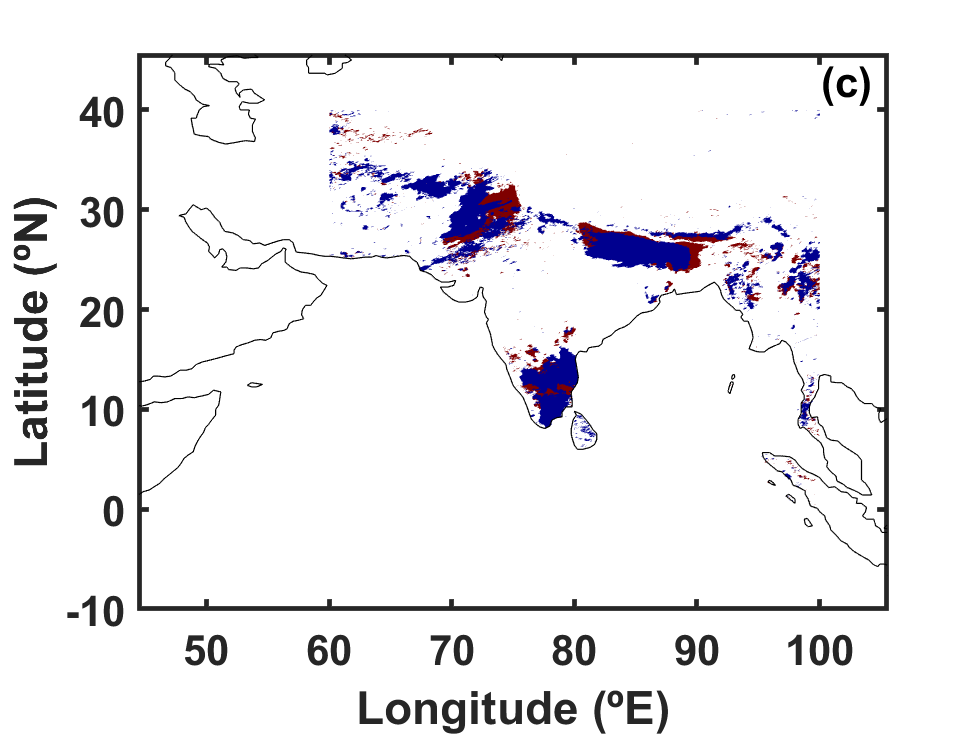 | (b)  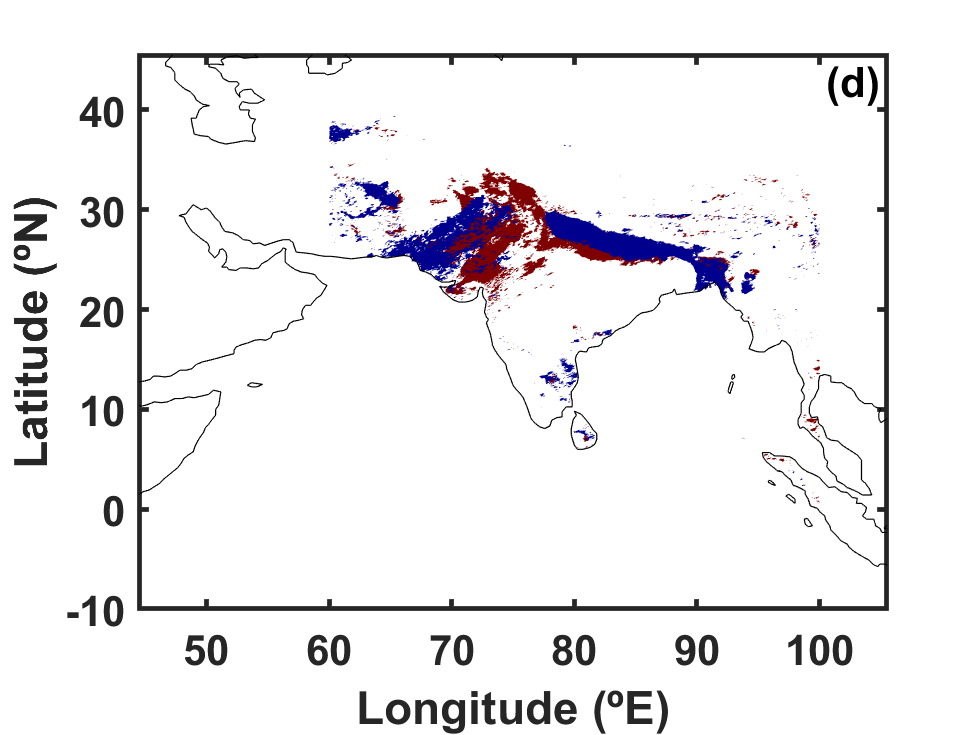 |
| --- | --- |
| (c)  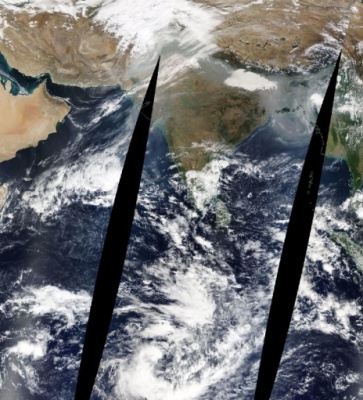 | (d)  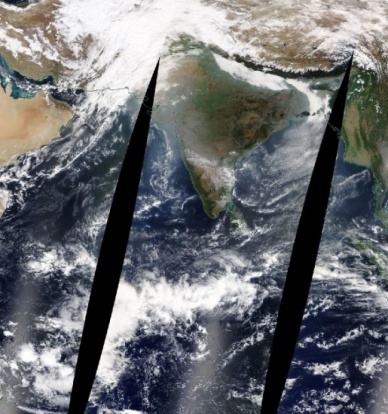 |
| (e)  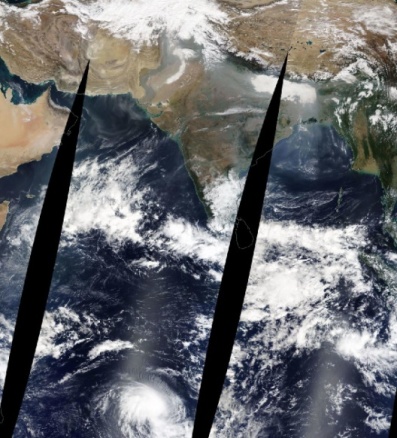 | (f)  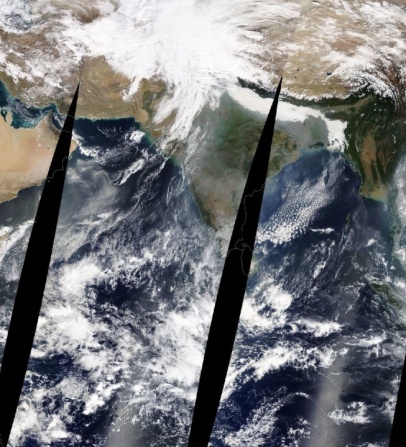 |

**Supplementary Figure S7**: (a) Enhancement of INSAT-3D retrieved fog from Dec 9 (in blue shaded area) to Dec 10, 2015 (red shaded area). (b) Enhancement of INSAT-3D retrieved fog from Jan 12 (in blue shaded area) to Jan 13, 2020 (red shaded area). Figure (a) & (b) are generated using MATLAB R2020a software available at <http://in.mathworks.com/products/matlab/>. (License no: 1103382).

MODIS image from <https://worldview.earthdata.nasa.gov/> showing the enhancement of fog from (c) Dec 9, 2015 to (e) Dec 10, 2015.

MODIS image from <https://worldview.earthdata.nasa.gov/> showing the enhancement of fog from (d) Jan 12, 2020 to (f) Jan 13, 2020.

| (a) Jan 24, 2020 from INSAT-3D  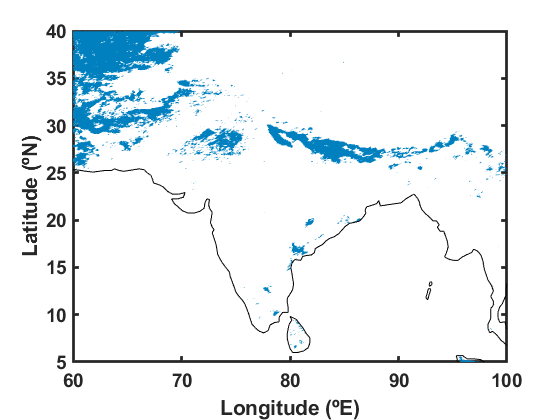 | (b) Jan 25, 2020 from INSAT-3D  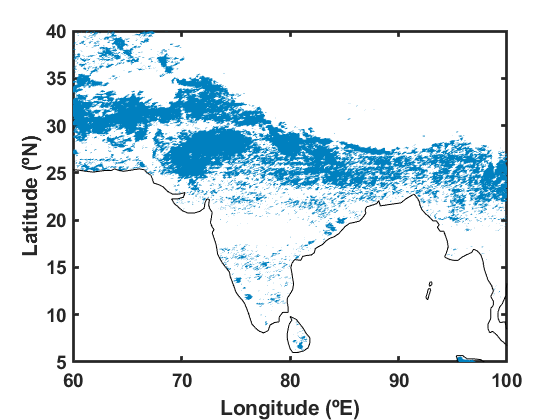 |
| --- | --- |
| (c) Jan 24, 2020 from MODIS  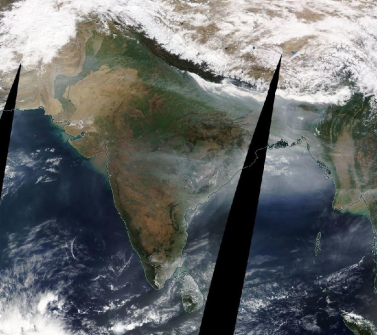 | (d) Jan 25, 2020 from MODIS  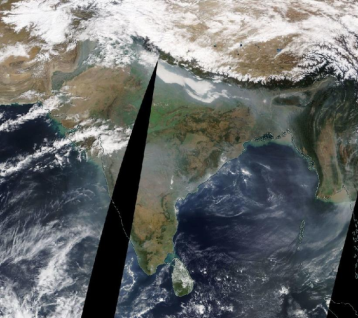 |

**Supplementary Figure S8**: The distribution of fog prevalence over IGP region on (a) Jan 24, 2020 and (b) Jan 25, 2020 from INSAT-3D, and using MODIS image from worldview on (c) Jan 24, 2020, (d) Jan 25, 2020. Figure (a) & (b) are generated using MATLAB R2020a software available at <http://in.mathworks.com/products/matlab/>. (License no: 1103382). Figure (c) & (d) are MODIS images from <https://worldview.earthdata.nasa.gov/> .

We acknowledge the use of imagery from the NASA Worldview application ([https://worldview.earthdata.nasa.gov](https://worldview.earthdata.nasa.gov/)), part of the NASA Earth Observing System Data and Information System (EOSDIS).

| 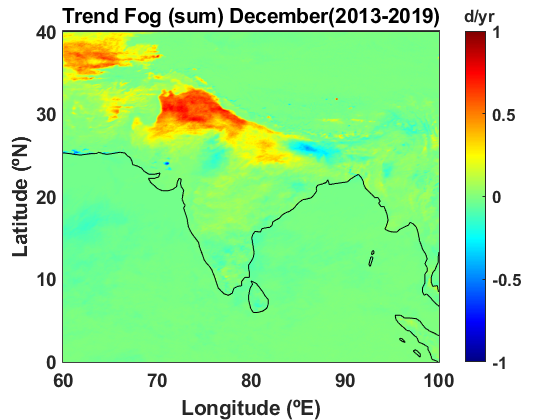 |
| --- |

**Supplementary Figure S9**: Spatial variation of trends in fog from satellite data for December during 2013 – 2019. Positive values show the regions, where fog is increasing and negative values indicate those, where fog is decreasing. Figure is generated using MATLAB R2020a software available at <http://in.mathworks.com/products/matlab/>. (License no: 1103382).

**Supplementary Table S1**: AR events characteristic parameters, for those shown in Figure 2(b). The length and width of each AR are calculated by checking if the IVT value surpassed a given threshold defined locally as the 85th percentile value. The Total IVT (TIVT) within the atmospheric river (AR) is calculated by summing all the IVT values that fall within the lateral edges of AR. The mean IVT of each AR and its standard deviation (SD) is calculated by considering those points that fall within the lateral edges of AR.

| **Date** | **Length,**  **km** | **Width,**  **km** | **Width-to-Length Ratio** | **Total IVT, Kg/s** | **Mean ± SD**  **IVT, Kg/m/s** |
| --- | --- | --- | --- | --- | --- |
| 30-12-2013 | 2,998 | 679 | 0.23 | 1.73 ×10^8^ | 236±190 |
| 20-01-2014 | 2,402 | 297 | 0.12 | 1.39 ×10^8^ | 275 ± 161 |
| 20-01-2015 | 1,497 | 739 | 0.49 | 1.91 ×10^8^ | 246 ± 168 |
| 09-12-2015 | 2,273 | 761 | 0.33 | 1.48 ×10^8^ | 223 ±143 |
| 09-12-2016 | 1,617 | 287 | 0.18 | 0.68 ×10^8^ | 232 ± 179 |
| 23-01-2017 | 1,910 | 935 | 0.49 | 2.16 ×10^8^ | 313 ± 236 |
| 17-12-2017 | 1,400 | 582 | 0.42 | 1.73 ×10^8^ | 245 ± 162 |
| 11-12-2019 | 2,508 | 415 | 0.16 | 1.65 ×10^8^ | 264 ± 203 |
| 06-01-2020 | 2,814 | 628 | 0.22 | 2.16 ×10^8^ | 366 ± 276 |
| 12-01-2020 | 1,997 | 420 | 0.21 | 1.77 ×10^8^ | 340 ± 249 |
| 24-01-2020 | 2,632 | 580 | 0.22 | 1.52 × 10^8^ | 247 ± 186 |
| 27-01-2020 | 2,387 | 483 | 0.20 | 1.27 × 10^8^ | 255 ± 195 |
| **Mean (and 95%confidence intervals)** | **2,203 ± 294** | **567 ± 110** | **0.27 ± 0.07** | **1.62 ± 0.23 x 10^8^** | **270 ± 26** |

**Supplementary Table S2**: Fractional increase in aerosol optical depth (AOD) and integrated water vapour (IWV) over IGP for an AR event on Jan 24, 2020 (before AR landfall) and Jan 25, 2020 (after AR landfall).

| Satellite (and time of the day) | Date | AOD over IGP  (Mean ± Standard Deviation) | Fractional increase in AOD |
| --- | --- | --- | --- |
| MODIS-Terra (1030hrs) | Jan 24, 2020 | 0.36 ± 0.25 | 60% |
|  | Jan 25, 2020 | 0.92 ± 0.66 |  |
|  | | | |
| MODIS-AQUA (1330hrs) | Jan 24, 2020 | 0.35 ± 0.26 | 53% |
|  | Jan 25, 2020 | 0.75 ± 0.47 |  |

| Date | IWV (Kg/m^2^) over IGP  (Mean ± Standard Deviation) | Fractional Increase in IWV |
| --- | --- | --- |
| Jan 24, 2020 | 8.84 ± 2.05 | 36% |
| Jan 25, 2020 | 13.91 ± 2.29 |  |

**Supplementary Table S3**: Fractional increase in integrated water vapour (IWV) over IGP for all the AR events. The IWV values on two consecutive days, which corresponds to before and after AR moved inland.

| AR event (date, month, year) identified on west-coast of India | IWV (Kg/m^2^) over IGP  (Mean ± Standard Deviation) | Fractional increase in IWV |
| --- | --- | --- |
| 30-12-2013 | 15.39±4.98  19.83±5.72 | 22% |
| 20-01-2014 | 11.64±2.45  16.36±7.35 | 29% |
| 20-01-2015 | 11.41±2.05  15.43±6.67 | 26% |
| 09-12-2015 | 19.32±2.40  23.69±1.72 | 18% |
| 09-12-2016 | 14.65±2.74  16.59±4.65 | 12% |
| 23-01-2017 | 15.17±2.04  17.33±3.82 | 12% |
| 17-12-2017 | 9.35±2.55  10.45±2.71 | 10% |
| 11-12-2019 | 20.37±2.77  26.21±2.06 | 22% |
| 06-01-2020 | 21.40±2.50  22.99±1.17 | 7% |
| 12-01-2020 | 15.63±2.01  18.49±3.55 | 15% |
| 24-01-2020 | 8.84±2.05  13.91±2.29 | 36% |
| 27-01-2020 | 20.08±2.27  25.55±3.68 | 21% |
| **Mean (and 95%confidence intervals)** | | **19 ± 5%** |

**Supplementary Table S4**: Fractional increase in aerosol optical depth (AOD) over IGP for all the AR events, where relatively cloud-free data exists. The AOD values on two consecutive days, which corresponds to before and after AR moved inland.

| AR event (date, Month, Year) on the west-coast of India | AOD over IGP  (Mean ± Standard Deviation) | Fractional increase in AOD |
| --- | --- | --- |
| 30-12-2013 | 0.88 ± 0.44  1.05 ± 0.52 | 16% |
| 20-01-2015 | 0.47 ± 0.26  0.51 ± 0.46 | 8% |
| 17-12-2017 | 0.50 ± 0.26  0.79 ± 0.63 | 37% |
| 06-01-2020 | 0.69 ± 0.43  0.91 ± 0.76 | 24% |
| 12-01-2020 | 0.49 ± 0.28  0.82 ± 0.61 | 40% |
| 24-01-2020 | 0.36 ± 0.25  0.92 ± 0.66 | 60% |
| 27-01-2020 | 1.15 ± 0.98  1.42 ± 0.71 | 19% |
| **Mean (and 95%confidence intervals)** | | **29 ± 13%** |
